# Impact of Noisy Labels on Dental Deep Learning—Calculus Detection on Bitewing Radiographs

**DOI:** 10.3390/jcm12093058

**Published:** 2023-04-23

**Authors:** Martha Büttner, Lisa Schneider, Aleksander Krasowski, Joachim Krois, Ben Feldberg, Falk Schwendicke

**Affiliations:** 1 Department of Oral Diagnostics, Digital Health and Health Services Research, Charité—Universitätsmedizin Berlin, 14197 Berlin, Germany; 2 ITU/WHO Focus Group AI4Health, Topic Group Dental Diagnostics and Digital Dentistry, CH-1211 Geneva 20, Switzerland

**Keywords:** artificial intelligence, machine learning, deep learning, computer vision, convolutional neural networks, calculus, digital imaging, radiology

## Abstract

Supervised deep learning requires labelled data. On medical images, data is often labelled inconsistently (e.g., too large) with varying accuracies. We aimed to assess the impact of such label noise on dental calculus detection on bitewing radiographs. On 2584 bitewings calculus was accurately labeled using bounding boxes (BBs) and artificially increased and decreased stepwise, resulting in 30 consistently and 9 inconsistently noisy datasets. An object detection network (YOLOv5) was trained on each dataset and evaluated on noisy and accurate test data. Training on accurately labeled data yielded an mAP50: 0.77 (SD: 0.01). When trained on consistently too small BBs model performance significantly decreased on accurate and noisy test data. Model performance trained on consistently too large BBs decreased immediately on accurate test data (e.g., 200% BBs: mAP50: 0.24; SD: 0.05; *p* < 0.05), but only after drastically increasing BBs on noisy test data (e.g., 70,000%: mAP50: 0.75; SD: 0.01; *p* < 0.05). Models trained on inconsistent BB sizes showed a significant decrease of performance when deviating 20% or more from the original when tested on noisy data (mAP50: 0.74; SD: 0.02; *p* < 0.05), or 30% or more when tested on accurate data (mAP50: 0.76; SD: 0.01; *p* < 0.05). In conclusion, accurate predictions need accurate labeled data in the training process. Testing on noisy data may disguise the effects of noisy training data. Researchers should be aware of the relevance of accurately annotated data, especially when testing model performances.

## 1. Introduction

Object detection is a computer vision technique which seeks to identify and label object instances within images by means of outlining rectangles, also called bounding boxes (BBs). Often, object detection models are trained in a supervised manner, with annotators labeling objects of interest by drawing BBs over them. In natural scenes of images this seems to be a relatively easy task for annotators as humans process such visual data without actively thinking about it. However, object detection has long moved beyond natural image scenes and has become an important component of medical image analysis [1,2].

Providing labels for medical data remains challenging. Besides datasets being smaller in comparison to open datasets of common objects (like cats or dogs) [3] experts are needed to label medical data. As labeling medical images is time consuming and as individual experts may miss certain findings often multiple experts are needed to label a dataset. These experts—even if not missing a finding—may introduce noise to a dataset during labeling: They may, if calibrated imperfectly, consistently label certain objects as too small or too large, for example, or be inconsistent in their labeling, e.g., one expert labels too small, one too large, and the third one perfectly right. The present study focuses on this type of label noise (other noise emanates from the discussed variability in accuracy, for instance).

Noisy labels in supervised learning are challenging researchers and data scientists. Supervised object detection models are trained by iterating through data. During the training process the model is being optimized to minimize the difference between the model predictions and the provided labels. Given noisy labels, the model may learn incorrect features to recognize an object. Different types of label noise have been described, e.g., inter-observer variability (inconsistent labels) and class-independent errors (consistent, e.g., too small or too big labels) [4]. The present study inspects the effects of on both aforementioned types of label noise.

In recent years first proposals have been developed to reduce the negative impact of noisy labels in machine learning, e.g., choice of loss function, data weighting or filtering of noisy labels [4,5]. Even though some approaches led to better model performance the impact of noisy labels remains not well described. Few studies have been conducted to investigate the influence of noisy labels on deep learning based object detection. The effect of additional, missing and shifted BBs was examined using SSD [6,7] and YOLOv3 [8,9] model architectures for tasks such as drone detection, demonstrating that especially missing BBs negatively impact model performance [9]. The beneficial effect of relabeling a noisy dataset with accurate labels was proven for tasks as maritime object detection using YOLOv5 [10,11]. In medicine noisy labels been explored even less often, e.g., for histopathological image analysis, demonstrating that too large labels negatively affect model performance. Further, inconsistent label sizes have been shown to be disadvantageous [12].

Demonstrating that noise is detrimental would underpin the relevance of accurate, consistent labeling. Automated detection of dental calculus on radiographs has so far not been studied but is relevant in the context of this study as calculus is represented by small irregular objects with oftentimes blurred boundaries, i.e., objects which are hard to label accurately. Bitewings are a type of dental radiograph that is used to visualize the coronal part of the posterior teeth. The main indication for bitewing radiographs is caries diagnosis. Clinically, the automated detection of calculus on radiographs could warrant further clinical examination and trigger certain therapies like professional tooth cleaning or scaling and root planning.

Our objective was to assess the impact of label noise on the performance of a state-of-the-art deep learning based object detection model for one particular problem: detection of dental calculus on bitewing radiographs. Our hypothesis was that both consistent and inconsistent noise significantly affects model performance. We further investigate the object detection models with explainable artificial intelligence (XAI) methods to visualize the impact of noisy labels on an exemplary model prediction.

## 2. Materials and Methods

### 2.1. Study Design

This study employed a commonly used deep learning based single-shot object detector: YOLOv5, which demands accurate labels for optimal performance. We employed a dataset of bitewing radiographs labelled for dental calculus by two calibrated experts using optimal (accurate) BBs. To simulate noise we first consistently increased or decreased the BBs sizes to generate consistently too small or too large labels. In a second step we increased and decreased only parts of the dataset, i.e., generated an inconsistently labeled dataset. We then explored the performance of YOLOv5 to detect calculus on these datasets and further employed methods of XAI to assess which image features were particularly relevant for the model’s decision when trained on differently noisy datasets. The waiver for informed consent is approved by ethics committee Charité—Universitätsmedizin Berlin. Reporting of this study follows the checklist for authors for artificial intelligence in dental research [13].

### 2.2. Dataset

Our dataset contained 4837 bitewings collected during routine care at a public university clinic in Berlin, Germany with radiographic machines from Dürr Dental SE (Bietigheim-Bissingen, Germany) and Sirona Densply Inc. (Bensheim, Germany). The prevalence of calculus on image level was 36.1%. Bitewings without calculus were excluded, resulting in 1746 included images from a German subpopulation with a mean [SD, min, max] age of 38.5 years [16.0, 4.8, 83.5], 51.0% males and 49.0% females. Two calibrated dentists, experienced in image analysis performed the labeling process. In the first cycle one expert labeled dental calculus using BBs with the aim of achieving the most accurate label (as small as possible but enclosing the whole object). A second expert checked all images in a second pass and controlled them once more resulting in the “accurate” base-case dataset. A comprehensive sample of available bitewings was used.

### 2.3. Simulating Noise

#### 2.3.1. Consistent Noise

**Figure 1:**
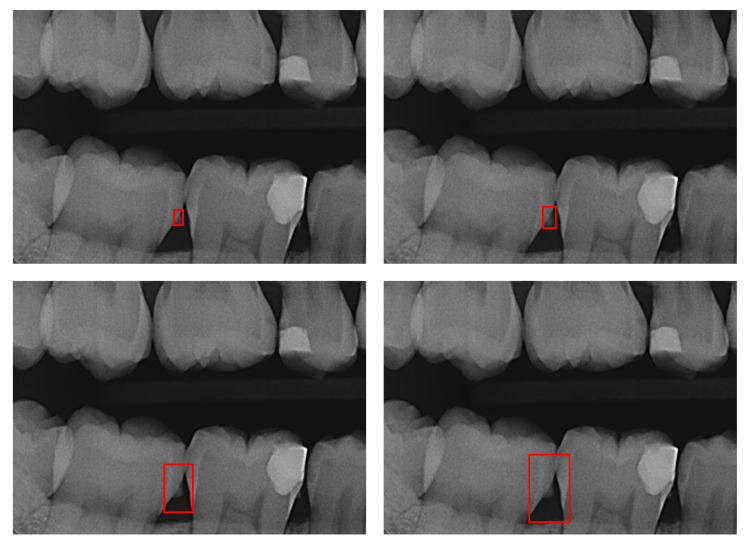
Example of simulated label noise: Correctly placed Bounding Boxes (BBs) outlining dental calculus were artificially increased and decreased to generate noise by multiplying the BB rectangle area with a factor *α*, resulting in consistently too small or too large BBs. Here, from left to right: *α* = 0.5, *α* = 1 (original), *α* = 5, *α* = 10.

To assess the impact of consistent label noise, the area of each BB was stepwise increased or decreased by a factor *α*. For this purpose, the center of the BBs was kept and the height and width were each multiplied by the square root of *α*. These labels will be referred to as manipulated labels below:

Original: x, y, h, w

$$\mathrm{Manipulated}:\;x,\;y,\;\sqrt{\alpha }\times h,\;\sqrt{\alpha }\times w$$

where x and y are the coordinates of the BB center, h the height and w the width of the BB. The experiments were conducted to the following *α* values: 0.1, 0.2, 0.3, 0.4, 0.5, 0.6, 0.7, 0.8, 0.9, 1, 2, 3, 4, 5, 6, 7, 8, 9, 10, 20, 30, 40, 50, 60, 70, 80, 90, 100 (Figure 1).

#### 2.3.2. Inconsistent Noise

A further experiment was performed to assess the impact of inconsistent noise. We simulated the behavior of three hypothetical experts, each labeling one third of the dataset (which may be the case for large datasets where it is impossible to have one expert label the full dataset). For the first third we kept the original BBs to represent an accurate labeler. For the second and third parts we increased and decreased the BBs respectively, to simulate too small and too large labeling. The manipulation of the labels was performed as described above with different deviations of too large and too small BBs. The deviation δ from the original annotation of the BB area was systematically increased from 0.1 to 0.9 in 0.1 steps. A δ of 0.1 means an α of 1.1 for manipulating the third of the too large labeler and an α of 0.9 for manipulating the third of the too small labeler, etc.

Noise was introduced to the overall dataset (including the validation and test dataset), while for the evaluation (see below) performance testing was performed on both noisy test data (as it can be expected that the test data would usually be drawn from an overall noisily labelled dataset as well) and accurate (non-noisy) test data to evaluate the “true” effect of noise, which may be disguised by testing on noisy data but also to gauge if only paying specific attention to labeling the test dataset would be an option.

Data preprocessing and manipulation was done with Pythons pandas library version 1.4.1 [14].

### 2.4. Model

In this study the state-of-the-art object detection model architecture “You Only Look Once” version 5 (YOLOv5) was employed [10]. YOLOv5 is a one-stage object detector architecturally similar to the single shot detector (SSD) [7] and RetinaNet [15]. YOLOv5 provides different sub architectures such as YOLOv5x, which is recommended for small objects. The model was pretrained on the Microsoft Common Objects in Context (COCO) dataset [3].

Each model was trained for up to 300 epochs, referring to a complete pass through the entire training dataset. The training process was stopped after 100 epochs without improvement on validation data (early stopping). Mosaic and right-left-flip data augmentation, techniques to increase the dataset with image modifications to build a more robust model, were applied. The model was optimized using stochastic gradient descent, an optimization algorithm to minimize the difference between the model predictions and the true labels (loss function). The number of images used in each iteration (batch size) was set to 16, while the step size to updates the model parameters (learning rate) was set to 0.01. Training was performed with an image resolution of 640 × 640. Five-fold cross validation was performed. Data was randomly split into separate training, validation and test sets of 60%, 20% and 20% respectively. For each fold the model for evaluation on the test set was selected based on the epoch with best performance on the validation set. All computations were performed on Nvidia A100 40 GB GPU.

### 2.5. Model Evaluation

Performance was evaluated using mean average precision with the intersection over union (*IoU*) threshold set to 50% (mAP50). *IoU* describes the overlap of the predicted BB (*pBB*) with the ground truth BB (*gBB*) in relation to the total area of unified BBs:

$$IoU=\frac{pBB\cap gBB}{pBB\cup gBB}$$

With the *IoU* being higher than the given threshold the object is counted as correctly detected (true positive—*tp*) or not (false positive—*fp*). Average precision (AP) is the weighted mean of Precision in the Precision-Recall-Curve—calculating Precision and Recall (sensitivity) with different model confidence thresholds.

Precision (*P*) describes what proportion of the detected calculus is truly dental calculus:

$$P=\frac{tp}{tp+fp}$$

Recall (*R*), in medical domain better known as sensitivity, describes how many of all existing concretions are detected:

$$R=\frac{tp}{tp+fn}$$

where *fn* are false negative/not detected BB. Mean average precision (mAP) is the mean of AP over all classes:

$$mAP=\frac{1}{N}\sum_{i=1}^{N}{AP}_{i}$$

where N is the number of classes. Since in this experiment only one class was considered, mAP is equivalent to AP. We nevertheless refer to mAP because it is the common metric to compare object detection models.

All models were examined for significant differences compared to the base-case model using non-parametric Mann-Whitney-U-test. *p*-values below 0.05 were considered statistically significant. The statistical analysis was performed with Pythons SciPy library version 1.9.0 [16].

### 2.6. Explainability

In order to interpret the model results an XAI method, namely SHapley Additive exPlanations (SHAP) based on Shapley Values was applied [17]. Shapley values capture the contribution of each feature to the prediction in comparison to the average prediction. Within object detection tasks these features are created by grouping pixels in the input images to form super pixels. The super pixels were subsequently included and excluded, and it was evaluated how much this affected the output of the model. The results were represented as heatmaps overlayed on the input image, i.e., the contribution of each super pixels to the model prediction was represented. Each image was divided into 400 (20 × 20) super pixels. The evaluation was performed 25 times per detection.

## 3. Results

### 3.1. Base-Case Model

**Figure 2:**
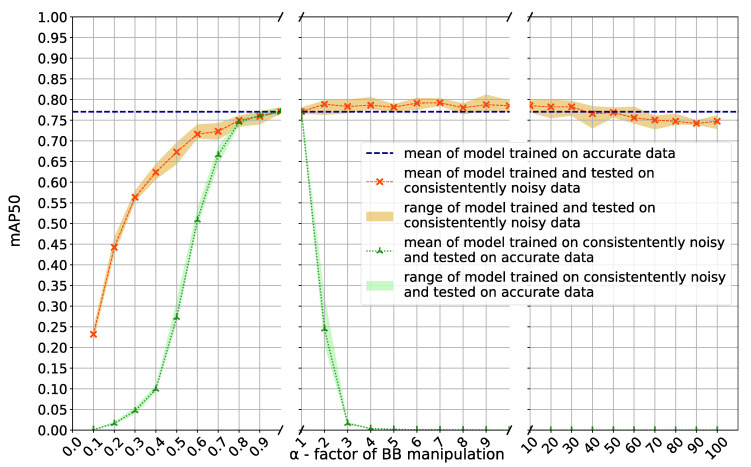
Performance (mean average precision with an intersection over union threshold of 50% (mAP50)) of calculus detection using deep learning models trained on consistently too small or too large BBs (*α*) when tested on consistently noisy test data (orange graph; mean and range of cross-validation mAP50 values) or accurate test data (green graph) compared with the model trained on accurate data (dashed blue line).

**Figure 3:**
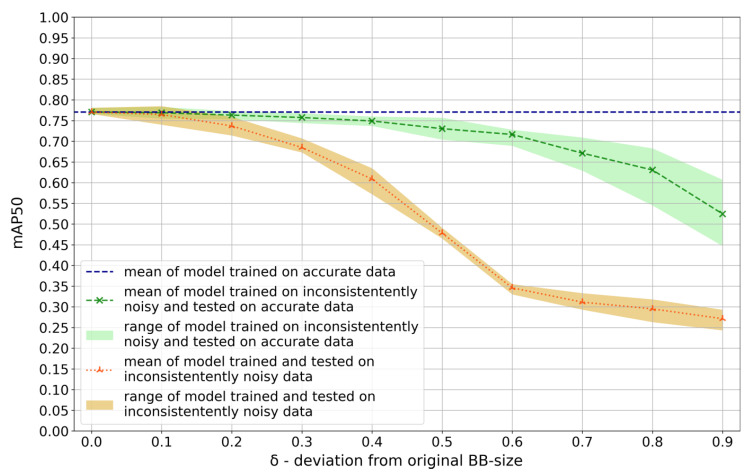
Model performance (mAP50) of models trained on inconsistently noisy data to simulate different annotators. Data was split into thirds. In one third of the data, the bounding boxes (BB) area was decreased, in one third kept constant, and in one third increased, respectively. δ specifies the deviation from the original size in both directions. Graphs show the mean and range values of models tested on such noisy data (orange graph) and tested on accurate data (green graph) compared with the model trained on accurate data (dashed blue line).

The model trained and tested on the base-case (accurate) dataset resulted in a mean [SD, min, max] mAP50 of 0.77 [0.01, 0.77, 0.78]. The base-case model is used as reference model and all models trained on noisy labels were compared against it (blue dashed line in Figure 2 and Figure 3).

### 3.2. Consistent Noise

Models trained on consistently smaller BBs showed significantly and escalatingly decreased performance when tested on noisy test data (orange graph in Figure 2; *p* < 0.05/Mann-Whitney). In contrast, increasing BB size up to *α* = 60 did not lead to significant changes in mAP50 (*p* = 0.15); moderate increase (e.g., BB size increased by *α* = 6) even led to a significant improvement (*p* = 0.02). However, if tested on accurate test data (green graph in Figure 2) the effect of consistently smaller BB was considerable once more while this time also consistently increased BB sizes detrimentally affected the model. Decreased BB sizes to α = 0.8 and increased to α = 2 already led to significant performance drops (*p* = 0.008 for both). Performance of models trained on consistent noisy labels were listed in Table S1 and Table S2 of the supplementary material.

### 3.3. Inconsistent Noise

When inconsistently noisy data (to simulate different annotators) was used, increasing noise had escalating detrimental effects when tested on inconsistently noisy data (orange graph in Figure 3). A δ of 0.2 or more caused a significant decrease to mean [SD, min, max] mAP50 0.74 [0.02, 0.71, 0.76] (*p* = 0.008). When the model was tested on accurate data (green graph in Figure 3) this effect was slightly attenuated; a δ of 0.3 or more resulted in a significant deterioration (*p* = 0.03), mean [SD, min, max] mAP50 of 0.76 [0.01, 0.74, 0.76]. Performance of models trained on inconsistent noisy labels were listed in Table S3 and Table S4 of the supplementary material.

### 3.4. Explainability

**Figure 4:**
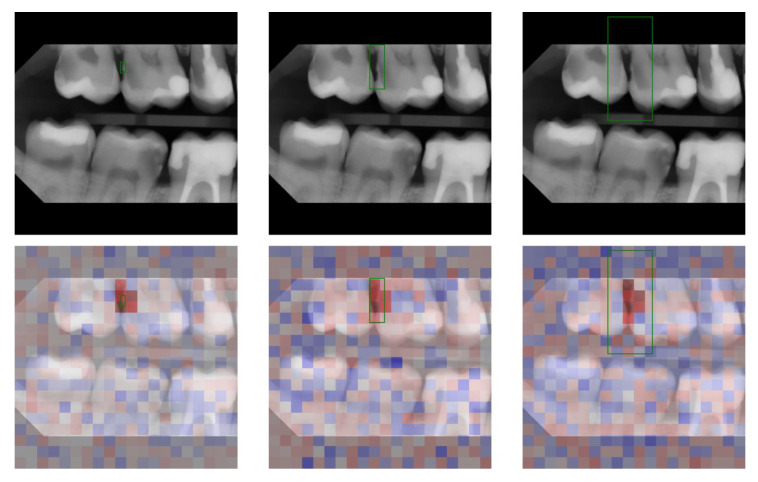
XAI evaluation. **First row**: Model prediction (green rectangle) trained on annotation with *α* = 1, *α* = 10, *α* = 100 (from **left** to **right**). **Second row**: SHapley Additive exPlanations (SHAP) heatmap where red values represent a positive contribution of the super pixel to the detection and blue values a negative contribution.

Figure 4 shows an evaluation of a detection using SHAP. Red values represent a positive contribution of the super pixel to the detection and blue values a contribution against it. Even with an area enlargement by a factor of 100, visible calculus still played an important role for the detection.

## 4. Discussion

Labeling medical data for deep learning is a complex and understudied aspect. Many studies published over the last years focus on developing models while basic research into understanding the impact of the labeling process and how to optimize it is scarce [2,18,19]. The present study assessed if consistent or inconsistent label noise detrimentally affects the performance of a deep learning model for a specific problem, calculus detection on bitewings. This exemplary task was chosen as calculus is highly prevalent and detecting it would be clinically relevant, but more so as labeling images for calculus is a task which may lead to noise given the discussed challenges. Our hypothesis that both consistent and inconsistent noise significantly affects the model performance needs to be accepted. Moreover, we demonstrated that the true performance of models trained on noisy data may not be reflected if the test data is similarly noisy; only on accurate test data the impact of label noise was fully reflected.

Our findings need to be discussed in detail. If the model was both trained and tested on consistently noisy data its performance remained moderately high for most datasets, indicating that inaccurate labeling and its impact on performance may be disguised if testing is performed on the resulting noisy data too. This was particularly true for larger BBs: When tested on noisy data the effect of enlarged BBs during training was absent for considerable time, even for massively increased sizes (*α* ≤ 60).

Evaluating the models with XAI methods as shown in Figure 4 gives us insights into possible causes: calculus remained most relevant for models prediction even when trained using large BBs; the super-pixels used to decide where calculus is present were near identical regardless of the BB size. Additional pixels included in the enlarged BB (*α* = 100) played a subordinate role for the prediction, likely as the standardization of the image (bitewings are similar to each other, which is different in comparison with natural scene images) allowed the model to learn the background and consequently ignore it successfully even if BBs were too large. The illustration of the predictions further show that the object (calculus) remained center of the predicted BBs. This reinforces the assumption that the model has learned the annotation error (consistently too large BBs). This phenomenon might be further explained by the learning process of neural networks: the model is optimized to predict BBs as close to the ground truth as possible. To increase the performance the objective thus becomes: (1) identification of the object of interest (calculus); (2) fit BB with dimensions to maximize area overlap with ground truth (e.g., consistently too large).

In contrast, if the BB were too small, we observed poor performance regardless of the chosen test (noisy or accurate). It can be assumed that given that calculus is already a very small object, models trained on too small BBs simply did not receive enough information to allow learning the features of calculus. Furthermore, with small objects, a small error already leads to the required *IoU* threshold not being reached. When tested on accurate data, the detrimental effect of noise on training success was demonstrated; researchers should pay special attention to consistent and accurate labeling of their test set to allow providing reliable information about the true accuracy of their model. This aspect of the results should be emphasized, since other studies dealing with noisy data tested their models solely on accurate data [4,9,12]. Testing on noisy data highlights the risk of performance obfuscation. It is especially relevant for medical applications where results are difficult to interpret and to compare due to a lack of standardized testing approaches and benchmarking datasets. In the medical domain deep learning models trained on noisy data are likely to be tested on a subset of the same distribution of data and therefore carry the same amount of noise.

In contrast, testing on noisy data may even lead to false conclusions, for example if a model was trained on accurate data and the (correctly) predicted BBs would not fit to the provided test data, leading to low performance metrics. A similar effect was demonstrated when dealing with inconsistent noise: Testing on accurate data showed no significant performance deterioration while δ ≤ 0.3, indicating that the model was capable of learning from inconsistent noisy labels. However, testing on noisy labels disguises this capability and already suggests a decrease in model performance when δ ≥ 0.2.

As discussed, several methods have been proposed to handle label noise through technical methods: a different loss function, measuring the difference between the model predictions and the desired output showed promising results when the underlying data set for a neural network contained noisy labels [4,20,21]. However, technical developments that aim to reduce the influence of label noise are often tested on clean benchmarking data sets. These data sets are currently not available in dentistry due to sensitive nature of the data and the associated data protection making the generation of a particularly clean test data set indispensable as demonstrated by the discussed results.

This study has a number of limitations. First, our goal was not to develop the best model for our specific problem; we did not aim to optimize performance by employing, for example, hyperparameter tuning but to understand the impact of noise in principle. Secondly, our trained models were not tested for generalizability while being developed using data from one German subpopulation only. Again, we accept this caveat in the context of our studys aims. Third, we assessed the impact of noise for one particular task, detecting calculus on bitewings. The effects on other modeling tasks like segmentation (where noisy labels are also likely) or other clinical problems (e.g., caries, apical lesion, periodontal bone loss detection) or images (other radiographs, photographs, histological data) may differ; we hence cannot claim transferability of our findings. Similarly only one model—YOLOv5—was employed; the effect of noise on other models may differ to some degree, as shown on histopathological images for cell segmentation [12].

## 5. Conclusions

Accurate predictions need accurate labeled data in the training process. Testing on noisy data may disguise the effects of noisy training data. Modelers should be aware of the relevance of accurately annotated data, especially when testing model performance, and users should scrutinize models accordingly for labeling quality.

## Data Availability

The weights of the trained models can be provided on request. Medical image data cannot be made available given data privacy reasons.

## Supplementary Materials

The following supporting information can be downloaded at: https://www.mdpi.com/article/10.3390/jcm12093058/s1, Table S1: Performance of calculus detection trained on consistently too large (*α* > 1) or too small (*α* < 1) bounding boxes when tested on accurate data; Table S2: Performance of calculus detection trained on consistently too large (*α* > 1) or too small (*α* < 1) bounding boxes when tested on consistent noisy data; Table S3: Model performance of models trained on inconsistently noisy data tested on inconsistent noisy data; Table S4: Model performance of models trained on inconsistently noisy data tested on accurate data.
