# Supplementary material for: Impact of Noisy Labels on Dental Deep Learning—Calculus Detection on Bitewing Radiographs
Source: J Clin Med. 2023 Apr 23;12(9):3058. doi: 10.3390/jcm12093058 (PMC10179289; doi:10.3390/jcm12093058)
Supplement: Supplementary file 1 [file jcm-12-03058-s001.zip › jcm-2315766-supplementary.pdf]

Supplemental Table S1: Performance (mean average precision with an intersection over union threshold of 50% (mAP50)) of calculus detection using deep learning models trained on consistently too large (α > 1) or too small (α < 1) bounding boxes when tested on accurate data.

α	mAP05	min	max	SD
0.1	0.0013	0.001	0.0015	0.0002
0.2	0.016	0.0132	0.0202	0.0026
0.3	0.0469	0.0392	0.0548	0.0059
0.4	0.099	0.092	0.108	0.0068
0.5	0.2726	0.258	0.313	0.0228
0.6	0.5084	0.494	0.536	0.017
0.7	0.6658	0.643	0.686	0.018
0.8	0.7448	0.738	0.753	0.0056
0.9	0.7618	0.753	0.77	0.007
1.0	0.7706	0.766	0.781	0.0062
2.0	0.2446	0.206	0.324	0.0462
3.0	0.016	0.0134	0.0195	0.0025
4.0	0.0039	0.0035	0.0045	0.0005
5.0	0.0021	0.0016	0.0026	0.0004
6.0	0.0009	0.0007	0.0011	0.0001
7.0	0.0009	0.0005	0.0012	0.0003
8.0	0.0005	0.0003	0.0005	0.0001
9.0	0.0003	0.0002	0.0005	0.0001
10.0	0.0002	0.0001	0.0005	0.0002
20.0	0.0	0.0	0.0001	0.0001
30.0	0.0	0.0	0.0	0.0
40.0	0.0	0.0	0.0	0.0
50.0	0.0	0.0	0.0	0.0
60.0	0.0	0.0	0.0	0.0
70.0	0.0	0.0	0.0	0.0
80.0	0.0	0.0	0.0	0.0
90.0	0.0	0.0	0.0	0.0
100.0	0.0	0.0	0.0	0.0

Supplemental Table S2: Performance (mean average precision with an intersection over union threshold of 50% (mAP50)) of calculus detection using deep learning models trained on consistently too large (α > 1) or too small (α < 1) bounding boxes when tested on consistent noisy data.

α	mAP05	min	max	SD
0.1	0.232	0.217	0.247	0.0111
0.2	0.4424	0.427	0.466	0.0171
0.3	0.5636	0.554	0.58	0.01
0.4	0.624	0.607	0.644	0.0151
0.5	0.673	0.644	0.696	0.02
0.6	0.7164	0.706	0.74	0.0136
0.7	0.723	0.703	0.743	0.0154
0.8	0.75	0.734	0.761	0.0104
0.9	0.7592	0.74	0.772	0.012
1.0	0.7706	0.766	0.781	0.0062
2.0	0.7888	0.763	0.798	0.0147
3.0	0.7834	0.767	0.8	0.0142
4.0	0.7864	0.776	0.806	0.0125
5.0	0.7816	0.771	0.789	0.0069
6.0	0.7916	0.775	0.804	0.0117
7.0	0.7924	0.785	0.803	0.0073
8.0	0.7802	0.764	0.791	0.0099
9.0	0.7878	0.773	0.812	0.0168
10.0	0.785	0.769	0.798	0.0118
20.0	0.7824	0.755	0.8	0.0174
30.0	0.7826	0.761	0.797	0.0132
40.0	0.7656	0.73	0.784	0.0244
50.0	0.7688	0.757	0.781	0.0096
60.0	0.756	0.742	0.783	0.0159
70.0	0.7504	0.728	0.761	0.0132
80.0	0.7476	0.739	0.766	0.011
90.0	0.7422	0.739	0.75	0.0047
100.0	0.7476	0.729	0.762	0.0127

Supplemental Table S3: Model performance (mAP50) of models trained on inconsistently noisy data to simulate different annotators tested on inconsistent noisy data. Data was split into thirds. In one third of the data, the bounding boxes (BB) area was decreased, in one third kept constant, and in one third increased, respectively. δ specifies the deviation from the original size in both directions.

δ	mAP05	min	max	SD
0.1	0.7646	0.74	0.785	0.0173
0.2	0.737	0.714	0.759	0.018
0.3	0.6848	0.673	0.707	0.0145
0.4	0.6088	0.572	0.635	0.0231
0.5	0.478	0.465	0.491	0.0111
0.6	0.3458	0.33	0.355	0.0109
0.7	0.3112	0.293	0.333	0.0156
0.8	0.295	0.263	0.318	0.0256
0.9	0.2712	0.243	0.293	0.0211

Supplemental Table S4: Model performance (mAP50) of models trained on inconsistently noisy data to simulate different annotators tested on accurate data. Data was split into thirds. In one third of the data, the bounding boxes (BB) area was decreased, in one third kept constant, and in one third increased, respectively. δ specifies the deviation from the original size in both directions.

δ	mAP05	min	max	SD
0.1	0.769	0.755	0.783	0.012
0.2	0.7632	0.753	0.773	0.0093
0.3	0.7574	0.744	0.768	0.0088
0.4	0.749	0.737	0.76	0.0095
0.5	0.7304	0.704	0.757	0.019
0.6	0.7164	0.689	0.728	0.0157
0.7	0.6712	0.629	0.709	0.0335
0.8	0.6306	0.545	0.683	0.0578
0.9	0.5244	0.447	0.607	0.0632
